# Surgical Use of Cocaine

**Published:** 1896-10

**Authors:** 


### Surgical Use of Cocaine.

We quote the following practical observations from the *Codex Medicus:*

1. The use of cocaine should not be abandoned because its irrational employment has produced deleterious results.

2. Always make a thorough physical examination of the patient before injecting the drug.

3. It should not be used in cases showing organic diseases of the brain, heart, lungs or kidneys, or in persons of neurotic diathesis.

4. Children bear it fully as well as adults.

5. The patient should always be placed in a recumbent position prior to its employment.

6. Constriction should be used whenever possible to limit the action of the drug to the desired area.

7. Use a freshly-prepared solution for each case.

8. Distilled water should always be employed, to which phenic, salicylic, or boric acid should be added.

9. A 2-percent solution has a better effect and is safer than solutions of greater strength.

10. Never inject a larger quantity than one and one-eighth of a grain when no constriction is used.

11. About the head, face and neck one-third of a grain should never be exceeded.

12. When constriction is possible, the dose may be as large as two grains.

13. Every slight physiological effect is not necessarily to be taken as cause for alarm.

14. Cocaine does have effect upon inflamed tissues.

15. In case alarming symptoms occur, use amyl nitrate, strychnine, digitalis, ether or ammonia.

To which we will add: Always use a chemically-pure product, free from isatropyl and cinnamyl-cocaine as well as other impurities, the presence or absence of which can be readily ascertained by the simple tests of the U. S. Pharmcopeia.—*American Therapist.*

